# Short-term exposure to stone minerals used in asphalt affect lung function and promote pulmonary inflammation among healthy adults

**DOI:** 10.5271/sjweh.4023

**Published:** 2022-06-30

**Authors:** Therese Nitter Moazami, Bjørn Hilt, Kirsti Sørås, Kristin V Hirsch Svendsen, Hans Jørgen Dahlman, Magne Refsnes, Marit Låg, Johan Øvrevik, Rikke Bramming Jørgensen

**Affiliations:** 1 Department of Industrial Economics and Technology Management (IØT), Norwegian University of Science and Technology (NTNU), Trondheim, Norway; 2 Department of Occupational Medicine, St. Olavs Hospital, Trondheim University Hospital, Trondheim, Norway; 3 Department of Public Health and Nursing, NTNU, Trondheim, Norway; 4 Clinical Research Ward, St. Olavs Hospital, Trondheim University Hospital, Trondheim, Norway; 5 Section of Air Quality and Noise, Department of Environmental Health, Norwegian Institute of Public Health (FHI), Oslo, Norway

**Keywords:** airway inflammation, human exposure chamber, non-exhaust emission, particulate matter, quartz diorite, rhomb porphyry, urban air pollution

## Abstract

**Objective:**

Stone minerals are a partially ignored environmental challenge but a significant contributor to urban air pollution. We examined if short-term exposure to two stone minerals – quartz diorite and rhomb porphyry – commonly used in asphalt pavement would affect lung function, promote pulmonary inflammation, and affect bronchial reactivity differently.

**Methods:**

Our randomized crossover study included 24 healthy, non-smoking young adults exposed to the stone minerals quartz diorite, rhomb porphyry, and control dust (lactose). Exposure occurred in an exposure chamber, in three separate 4-hour exposure sessions. Fractional exhaled nitric oxide (FeNO) and lung function were monitored before exposure, then immediately following exposure, and 4 and 24 hours after exposure. In addition, methacholine was administered 4 hours following exposure, and exhaled breath condensate (EBC) was collected before exposure, then immediately and 4 hours after exposure. EBC was analyzed for pH, thiobarbituric acid reactive substances (TBARS), intercellular adhesion molecule 1 (ICAM-1), interleukin-6 (IL-6), IL-10, P-Selectin, surfactant protein D (SP-D), and tumor necrosis factor-α (TNF-α).

**Results:**

Our results showed significantly elevated concentrations of FeNO after exposure to quartz diorite compared to rhomb porphyry, suggesting that quartz diorite is more likely to trigger pulmonary inflammation after short-term exposure. Moreover, short-term exposure to rhomb porphyry was associated with a modest but statistically significant decline in forced vital capacity (FVC) compared to quartz diorite.

**Conclusion:**

These results emphasize that using stone material in asphalt road construction should be reconsidered as it may affect lung inflammation and lung function in exposed subjects.

Inhalation of particulate matter (PM) causes immunological reactions in the airway’s epithelium, producing local and systemic effects. In addition to the physical properties of the particles (size, particle number, and surface area), PM chemical composition is a critical determinant of the adverse health effects caused by exposure (1, 2).

Inflammation is a nonspecific, complex reaction involving various mechanisms to protect the body against pathogens and prevent and repair tissue damage (3). Pathophysiological reactions arise from multiple mechanisms (4), involving an inflammatory response. One marker of inflammation in the lungs is nitric oxide (NO) in exhaled air, formed through the oxidation of the amino acid -arginine by nitric oxide synthetase (NOS) and activated by inflammatory cytokines (5). Increased NO levels can be measured in exhaled air (FeNO) (6). Another non-invasive method for detecting biomarkers of lung oxidative stress and inflammation, mainly from the lower respiratory tract, is the analysis of different markers in exhaled breath condensate (EBC) (7).

Airway hyperreactivity (AHR) results from inflammatory reactions in the airway. AHR can be measured by various means, such as the methacholine challenge test, in which airway smooth muscle muscarinic receptors are activated to induce bronchoconstriction (8, 9).

Although the research and regulatory policies on ambient PM have focused mainly on combustion-derived PM, especially diesel exhaust particles (DEP), interest has now shifted to non-exhaust emissions, including road pavement abrasion and wear from tires and brakes (10). Some types of asphalt contain large amounts of highly reactive quartz, while others contain mainly feldspar, which has been considered less reactive (11). Thus, the chemical compositions of stone minerals are essential in assessing both occupational exposure, and public health effects from road abrasion and sandstorms, such as Asian dust, and other PM_2.5_ pollution episodes (12, 13). Whereas quartz and asbestos particles/fibers are known to trigger specific lung diseases (14), less is known about the potential health effects of other mineral particles (11). Notably, the role of stone material in pro-inflammatory responses has mainly been examined in cultures of lung epithelial cells (11, 15, 16). To extend this line of inquiry, we initiated a clinical study involving short-term exposure to two stone materials, quartz diorite and rhomb porphyry, used in road pavement. Using a human exposure chamber, we have observed limited systemic inflammatory and pro-coagulant responses following short-term exposure to quartz diorite in 24 healthy young volunteers (17, 18). In this part of the study, we aimed to determine how inhalation of these stone materials influences pulmonary inflammation, lung function, and airway reactivity.

## Methods

### Study population

An information letter with an invitation was distributedto recruit volunteers (university students), and subsequently 42 potential participants were invited to an information meeting about the study, of which 1 potential participant withdrew due to allergy. The remaining participants were invited for a pre-investigation session performed by a medical doctor. The volunteers were asked about their health, lifestyle, medication, and possible lactose intolerance. In particular, it was inquired about previous and present inflammatory diseases, and only candidates without any known such illnesses or symptoms were included in this study. Finally, 24 non-smoking, healthy university students were recruited. The distribution of females and males was 14:10, with a mean age of 23.6 [standard deviation (SD) 2.1] years and a mean body mass index (BMI) of 24 (SD 4.4) kg/m^2^. The participants were asked to avoid heavy exercise and alcohol 36 hours before each exposure session and to avoid certain types of food rich in nitrates in the morning before exposure considering nitrate-rich food has been found to influence FeNO substantially (19).

The participants were assigned to groups of four and exposed to the two stone minerals quartz diorite, rhomb porphyry, and lactose in random order across three separate exposure sessions. Each exposure session lasted 4 hours. Lactose was used as presumably inert control dust. The participants entered the exposure chamber at 30-minute intervals, in which the first participant entered the chamber at 09:00 hours, and the final participant entered the chamber at 10:30 hours. During exposure, the participants were seated at a table in the middle of the chamber. The participants changed seats every hour to reduce the variability in exposure caused by location within the room. The exposure to each type of dust was double-blinded, which was maintained until statistical analyses were performed. Three-week intervals separated the exposure sessions to avoid hang-over effects. All participants received oral and written information about the clinical trial and signed informed consent before enrolment. The Regional Ethics Committee approved the study (approval no. 260381), project grant no. 260381.

### Stone materials

**Figure 1:**
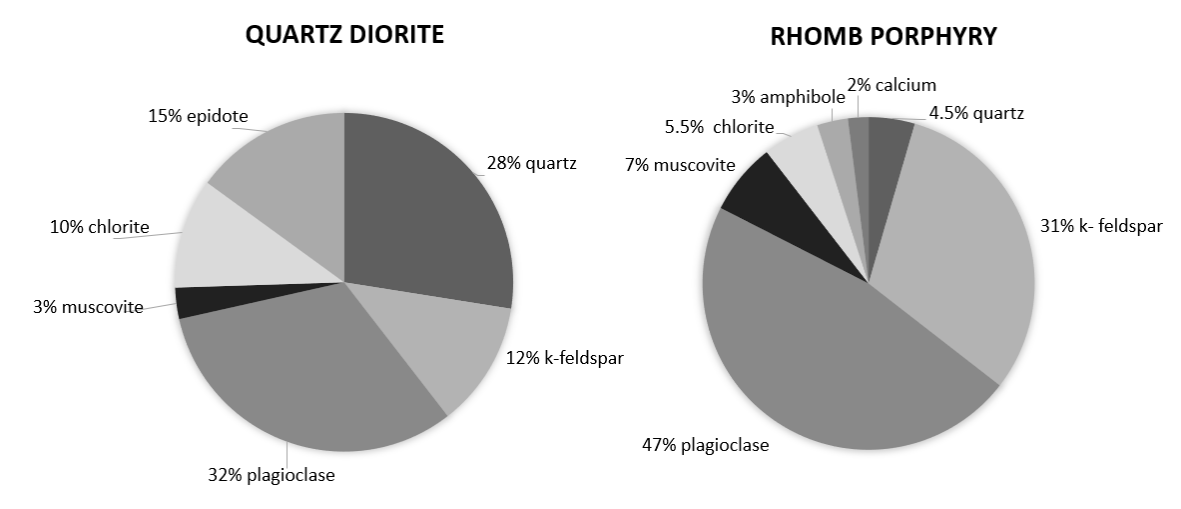
Mineral composition, in percent, of quartz diorite and rhomb porphyry.

The quartz diorite and rhomb porphyry materials were crushed into the desired fractions using the Los Angeles method (20), and the mineral compositions were determined using X-ray diffraction (XRD) analyses. The XRD results are shown in figure 1.

The particle size distribution of the three dust types was measured with an Aerodynamic Particle Sizer type 3321 from TSI. Quartz diorite showed a dominating mode of 0.84 μm, a geometric mean (GM) of 1.20 μm, and a geometric standard deviation (GSD) of 0.84 μm. Rhomb porphyry had an almost identical particle size distribution with a dominating mode of 0.78 μm, a GM of 1.18 μm, and a GSD of 1.75 μm. In comparison, lactose showed a more even size distribution, with dominating mode of 2.13 μm, a GM of 1.98 μm, and a GSD of 1.98 μm.

### Assessing exposure

The dust generation and exposure chamber have been described previously (18) and are not repeated here. The concentrations of total dust (stationary samples) and respirable dust (personal samples) were measured gravimetrically using a cyclone (SKC aluminum respirable cyclone) equipped with a cassette (SKC SureSeal 3-piece 37 mm) containing polyvinyl chloride (PVC) filters (SKC PVC filter 37 mm 5.0 μm). The filters were coupled to a sampling pump (SKC AirChek 3000 or CasellaTuff personal air sampler), calibrated to deliver a flow rate of 2.5 L/min for 4 h (respirable dust) and 2.0 L/min for 4 h (total dust). The dust supplied to the chamber was calibrated not to exceed the Norwegian occupational exposure limit values (OEL), representing the maximum average airborne concentration of a toxic substance over an 8-hour shift. The Norwegian OEL of total dust and respirable dust is 10 mg/m^3^ and 5 mg/m^3,^ respectively.

### Clinical investigations

For each exposure setting, lung function and FeNO in exhaled breath were measured before exposure (baseline readings) as well as immediately, 4 hours, and 24 hours after exposure. Additionally, EBC was collected at baseline, immediately, and 4 hours after exposure, whereas the methacholine test was performed just once 4 hours after exposure.

Experienced personnel performed pirometry testing using the Spirare computerized spirometer (Spirare sensor (SPS330) SN:217665 and SN:201491) by following the European Respiratory Society guidelines (21). The spirometer was calibrated before each test. The spirometry test was repeated three times for each participant to produce good flow-volume curves, again following the guidelines. For the statistical analyses, the best recordings of forced vital capacity (FVC), forced expiratory volume in one second (FEV_1_), and peak expiratory flow (PEF) were used. Measurements of forced expiratory time (FET), forced expiratory flow between 25–75% of FVC (FEF 25–75%), and the FEF measured at 75% of FVC (FEF 75%) from the maneuver that had the highest sum of FEV_1_ and FVC were also recorded.

EBC was collected while participants were seated upright and wearing a nose clip, using EcoScreenTM. The EBC was collected according to standard protocol with 10 minute tidal breathing. Condensate fluid was collected and divided into aliquots in vials coated with 1% bovine serum albumin (BSA) and immediately frozen at -20°C. At the end of the exposure session, the aliquots were stored at -80°C before being sent for analysis. All the condensate fluid samples were analyzed for pH using a pH meter (SevenEasy, Mettler Toledo GmbH, Switzerland) with a Sentix Mic pH electrode (WTW, by Xylem Analytics, Germany). The EBC samples were again frozen at -80 °C and then lyophilized in a freeze-dryer (Leybold-Heraeus GT2, Germany). The freeze-dried powder was resuspended in 200 μl water/diluent and immediately analyzed for levels of thiobarbituric acid reactive substances (TBARS) using a Parameter™ TBARS assay (RnD Systems, Inc, Biotech, USA) and BioTek Epoch 2 microplate reader (BioTek Instruments, Inc., USA) with Gen5 software ver. TS 2.09.1 (BioTek, USA). The assay protocol was followed, although the samples and reagents were applied at half their recommended volumes. Our assay had detection limits of 0.079 μM (range 0.05–0.11 μM); the lowest standard was 0.26 μM. EBC levels of intercellular adhesion molecule-1 (ICAM-1), interleukin (IL)-6, IL-10, P-Selectin, surfactant D (SP-D), and tumor necrose factor -α (TNF-α) were analyzed using Luminex® assays (RnD Systems, Inc., Biotechne, SA) and the Bio-Plex 200 instrument with Bio-Plex Manager software ver. 6.2. (Bio-Rad, USA). The levels of all these mediators in the Luminex assays were below their detection levels, and close to the blank values according to selected samples after reconstituting the samples in water or Dulbecco phosphate buffer solution (Gibco, UK) supplemented with 1% BSA (Sigma A9647, Sigma Aldrich, USA).

The methacholine inhalation test was performed using Pred. Module GLI/ECCS/Gulsvik. After the first FEV_1_ registration, the participants inhaled normal NaCl (18 mg), followed by inhalation of increasing doses of methacholine, starting from 0.05 mg and going up to 0.10, 0.30, 0.60, then 0.95 mg, resulting in corresponding cumulative dosages of 0.05, 0.15, 0.45, 1.05, and 2.0 mg, respectively. The responses were measured by registration of FEV_1_ before inhalation and at 30, 90, and 180 seconds after each inhalation. The inhalations were discontinued when a fall in FEV_1_ of 20% or more below the lowest post NaCl value was observed. FeNO was measured before spirometry and without a nose clip using the validated NIOX VERO, and following its investigator user manual, a constant sampling flow rate of 50 ± 5 mL/s was applied (22).

### Statistical analysis

According to the Shapiro Wilk test, some of the markers of lung function and FeNO were skewed and continued to be so after log-transformations and after transformations to percent change from baseline. No statistical difference in baseline readings was observed for the lung function indexes before exposure to the three exposure materials. Thus, for descriptive purposes, these variables are presented graphically using the median percent change from baseline. FeNO baseline readings for the exposure materials showed significant differences, so FeNO levels have been presented graphically using median values and interquartile ranges (IQR).

The absolute change in the pH in EBC from baseline to after exposure was normally distributed. The pHs of pairs of materials were compared using paired t-tests. The percent and absolute changes after each dose of methacholine were used in the statistical analyses. These values were normally distributed, and paired t-tests, coupling two materials simultaneously, were used to test for significant differences.

When applying the linear mixed-effect model (LMM), the observations obtained from each participant were assumed to be correlated, and observations from different participants were assumed to be independent. It was also assumed that the errors had a normal distribution with mean zero and constant variance (23). After log-transformation, these model assumptions were met for FeNO, FVC, and FEV_1_. The Sandwich Huber estimator was used to relax model assumptions for the remaining variables since their residuals were slightly skewed. A Bonferroni posthoc test was applied to the estimated marginal means for the LMM. One model was built for each variable, with baseline readings interpreted as a covariate. Considering that the participants were used as their own control, adjusting for variables such as gender and age was unnecessary. The random intercept and slope for the participants were included. Before the study started, a power calculation was performed, with a two-sided 5% significance level, based on different mean values for 24 subjects. A mean change of at least 1.25, with a standard deviation of 1/3 of this value, was assumed to be sufficient to achieve statistically significant differences for at least some of the included parameters. All statistical analyses were performed using SPSS version 28 (IBM Corp, Armonk, NY, USA) and STATA version 17 (StataCorp, College Station, TX, USA).

## Results

### Exposure concentrations

The exposure concentrations of quartz diorite and rhomb porphyry in the exposure chamber were reasonably similar, with median achieved concentrations of respirable particles (personal samples) of 5.4 and 5.6 mg/m^3^, respectively. The total dust concentrations of the minerals measured gravimetrically (stationary samples) were 20.8 and 21.9 mg/m^3^, respectively_._ The concentrations of respirable particles and total dust achieved during exposure to lactose were lower (0.3 mg/m^3^ and 5.7 mg/m^3^, respectively). Nevertheless, the exposure chamber looked and felt dusty during exposure to the lactose powder, and neither the researchers nor the volunteers could determine which material was in the exposure chamber.

### Fractional exhaled nitric oxide (FeNO)

In table 1, the antilog of the log-transformed estimate of fixed effects (β) from the LMM is shown for FeNO and some other outcomes that are presented later. All results are adjusted for baseline readings and sampling time. Lactose is used as a reference and assigned a value of 1. For the FeNO, the estimate for quartz diorite is 1.19, and for rhomb porphyry, 1.02, meaning that FeNO showed the highest increase after quartz diorite exposure. After exposure to quartz diorite, the observed increase was significantly higher than those observed for rhomb porphyry and lactose.

**Table 1:** Anti-log values from the mixed-effects model for fractional exhaled nitric oxide (FeNO) and lung function by exposure material. Estimates were adjusted for baseline values. [FEV_1_=forced expiratory volume in one second; FVC=forced vital capacity; FEF=forced expiratory flow; CI=confidence interval.]

Estimate	FeNO		FEV1		FVC		FEF 25%	
			
	β	95 % CI	β	95 % CI	β	95 % CI	β	95 % CI
Fixed effects								
Intercept	11.33 ^a^	0.96–1.18	1.29 ^a^	1.27–1.31	1.31 ^a^	1.69–1.93	3.66 ^a^	3.18–4.28
Lactose	1		1		1		1	
Quartz diorite	1.19 ^a^	1.10–1.26	1.00	0.99–1.01	1.00	0.99–1.01	1.00	0.97–1.02
Rhomb porphyry	1.02	0.94–1.10	0.99 ^a^	0.98–1.00	0.99 ^a^	0.98–1.00	0.98	0.96–1.00

a Significant at 0.05 level

**Figure 2:**
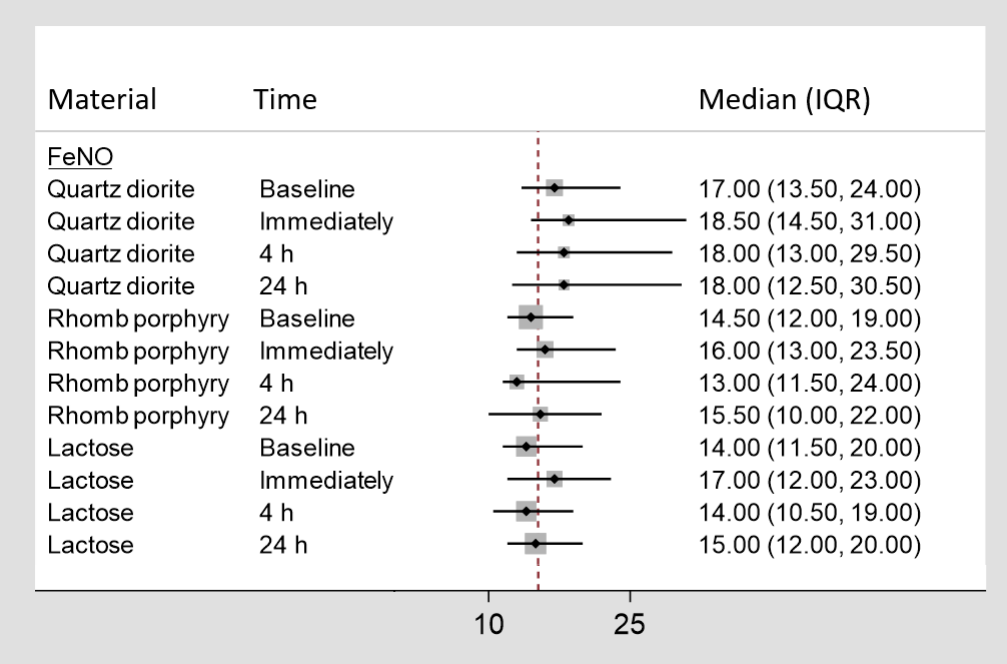
The medians (in ppb) and 25^th^ and 75^th^ percentiles [interquartile range (IQR)] for fractional exhaled nitric oxide (FeNO). The dotted line represents the average value.

In figure 2, the median measured concentration of FeNO (ppb), along with the 25^th^ and 75^th^ percentiles, for each exposure time and material are listed separately.

As shown in figure 2, an increase in the median was observed from baseline and immediately following exposure to all three exposure materials.

According to the LMM, after adjusting for baseline values the estimate for quartz diorite reached statistical significance (P=0.02). According to the posthoc test, the difference observed in FeNO following exposure to quartz diorite was statistically significantly different from those observed for lactose (P=0.00) and rhomb porphyry (P=0.00).

### Lung function

**Figure 3:**
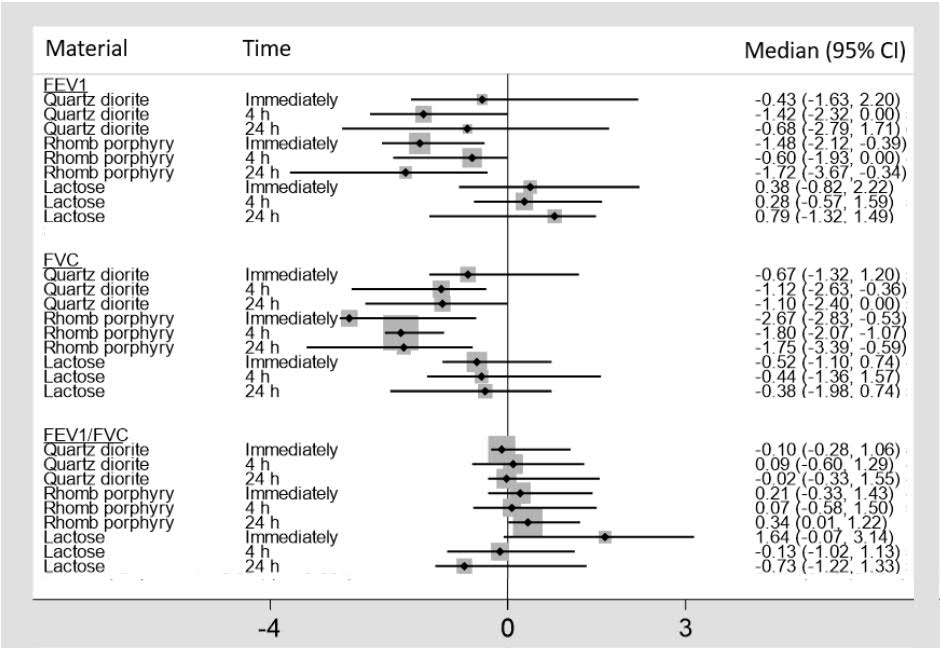
Median percent change, with 95% confidence intervals (CI), from baseline for forced vital capacity (FVC), forced expiratory volume in one second (FEV_1_), and the FEV_1_/FVC ratio.

**Figure 4:**
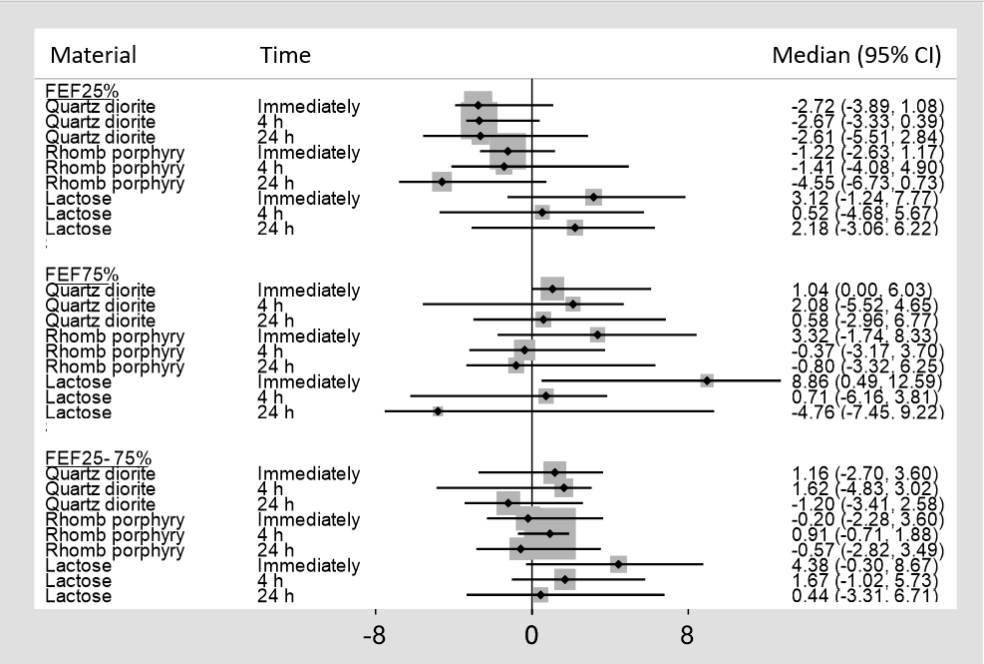
Median percent change, with 95% confidence intervals (CI), from baseline for forced expiratory flow (FEF) 25%, FEF75%, and FEF25–75%

In figures 3 and 4, the median percent change from baseline, along with its 95% confidence interval (CI), is shown for the various lung function indexes, and, in table 1, the antilogs of the log-transformed estimates of fixed effects (β) from the LMM are shown for FEV_1_, FVC, and FEF25%. The remaining lung function variables are not included in table 1, as none of the estimates for the materials were significant.

As shown in figure 3, both FVC and FEV_1_ show a modest decline immediately following exposure to quartz diorite, while they continue to decrease four hours after exposure. Twenty-four hours after exposure to quartz diorite, FVC stays at approximately the same level as observed four hours after exposure, while FEV_1_ begins to normalize again. This pattern was not observed following exposure to rhomb porphyry, where a significant decline in both FVC and FEV_1_ was observed immediately after exposure. Twenty-four hours after exposure to rhomb porphyry, the most significant drop in FEV_1_ was observed (-1.72%) while FVC started to normalize again. According to the posthoc test, the difference in FVC for quartz diorite and rhomb porphyry was statistically significant. The differences in FEV_1_ observed between rhomb porphyry, and lactose reached statistical significance (P=0.04), while the difference between rhomb porphyry and quartz diorite was insignificant (P=0.11). For the FEV_1_/FVC ratio, no statistically significant differences were observed for the three exposure materials.

As shown in figure 4, a median percent decrease of FEF25% by 4.55% was observed 24 hours after exposure to rhomb porphyry. In comparison, a median percent reduction of 2.61% and an increase of 2.18% were observed 24 hours following exposure to quartz diorite and lactose, respectively. The differences observed in FEF 25%, FEF% 75 and FEF 25–75% among the three exposure materials did not reach statistical significance. Finally, no significant differences were observed in FEV, PEF, and FET following exposure to the three exposure materials (data not shown).

### Methacholine challenge test

Defined by ≥20% decrease in breathing ability compared to baseline, 23% of the methacholine challenge tests were positive, while seven volunteers had a positive methacholine challenge after all exposure sessions. However, no association was found between the methacholine challenge test and the three exposure materials (data not shown).

### Exhaled breath condensate measurements (EBC)

In addition to FeNO, EBC was also used to assess airway inflammation. The EBC samples were first analyzed for potential pH changes. Using the paired t-test, no significant difference was observed in the pH readings from EBCs following exposure to either exposure material in our study (data not shown).

TBAR in EBC can measure oxidative stress in the bronchial epithelium. In an initial analysis, TBAR values were below the detection level. To increase the sensitivity, the EBC samples were freeze-dried and resuspended in 200 μl water, thus increasing the concentration by approximately 10x. Notably, our analysis showed that approximately 70% of the freeze-dried samples still gave TBAR values below the detection level, thus making it less meaningful to calculate the responses (data not shown). In the resuspended freeze-dried samples, ICAM-1, IL-6, IL-10, P-selectin, SP-D, and TNF-α also showed values below detection levels (not shown).

## Discussion

To our knowledge, this is the first study to examine early, short-term changes in pulmonary inflammatory reactions, lung function, and bronchial reactivity following exposure to different types of stone minerals used in asphalt. In this study, a statistically significant increase in FeNO was observed following exposure to quartz diorite, with a bit of delayed effect, while a non-significant decline was observed in FEV_1_, FVC, and FEF25%. No significant change was observed in FeNO following exposure to lactose and rhomb porphyry. However, a statistically significant decline in FEV_1_ and FVC following exposure to rhomb porphyry was observed, and for FVC, the difference observed between quartz diorite and rhomb porphyry reached statistical significance. Measurements of FeNO have been reported to have excellent reproducibility and high sensitivity and specificity in diagnosing airway inflammation (6). The FeNO levels in exhalations have previously been reported upon PM exposure. In a recent randomized crossover study of exposure to PM_2.5_ pollution episodes amongst healthy young adults, FeNO was significantly increased (13). A recent meta-analysis found that for each 10 μg/m^3^ increase in PM_10_ and PM_2.5,_ there were statistically significant increases in FeNO of 3.2% and 2.3%, respectively (24).

The potential for acute exposure to mineral particles to increase FeNO levels has been examined to a lesser degree. In a cross-sectional study including 11 mason workers and 21 electricians, the effect of silica (quartz) exposure on FeNO level was insignificant (25). In another study of 94 silica-exposed workers and 35 healthy volunteers, silica exposure was associated with significantly higher levels of alveolar NO, indicating inflammatory effects of silica in peripheral lung areas (26). Increased alveolar NO concentrations have also been found in patients exposed to asbestos, indicating an inflammatory process at the alveolar level (27). The difference observed in FeNO levels between quartz diorite and rhomb porphyry could be attributed to chemical composition, as previous studies have documented elevated FeNO levels after exposure to silica dust (26). Notably, FeNO changes have exhibited considerable diurnal variation caused by various factors (6, 28). The diurnal variations were controlled for in our study as the measurements were done simultaneously on each exposure day.

Exposure to air pollution is a known risk factor for decreases in lung function in children and adults (29, 30). In previous cross-sectional studies of short-term exposure to PM amongst healthy adults, an association between PM exposure and declines in lung function indexes has been found (31, 32). In a recent randomized, double-blind crossover study, in which 20 healthy young volunteers were exposed to PM_2.5_ mean concentrations of 37.8 μg/m^3^ and filtered air in 4-hour exposure sessions, the investigators observed decreases in FEV_1_, PEF, and FEV_1_/FVC of 0.8%, 1.8%, and 1.2%, respectively (33).

The effects of mineral particle exposure have been primarily examined in long-term occupational settings. For example, a meta-analysis of long-term exposure to α-quartz in the workplace showed marked and significant reductions in FEV_1_ and FEV_1_/FVC (34). In our randomized, double-blind crossover study, a decline in FEV_1_ was observed following exposure to quartz diorite and rhomb porphyry, while it increased following exposure to lactose. Notably, the present findings align with a previous study showing a decline in FEV_1_ of 0.5% following exposure to cooking fumes (35).

In our study, no association between the outcome of the methacholine challenge tests and the three exposure materials was observed. As the stone materials used in our study did not affect the reactivity of smooth muscle cells in the airways in healthy subjects differently, we think it is fair to assume that the methacholine challenge test may not be suitable for this type of investigation.

In a previous study investigating the effects of short-term exposure to diesel exhaust among people with asthma in an urban roadside environment, a drop in EBC pH was observed (36). However, no difference was observed between the three exposure materials in the present study for pH measured in EBC.

### Comparing the clinical studies to in vitro studies

While the quartz diorite used in the present study mainly consisted of plagioclase (32%) and quartz (28%), the rhomb porphyry consisted mainly of the feldspar minerals plagioclase (47%) and K-feldspar (31%). A recent in vitro study using models of lung cells (bronchial epithelial cells; HBEC3-KT and THP-1 macrophages) showed that quartz diorite was more potent than rhomb porphyry in inducing the pro-inflammatory cytokines IL-8 and TNFα in THP-1 macrophages. In contrast, no significant difference was observed in the HBEC3-KT cells exposed to the two stone materials. It should be emphasized that the overall potencies of these two stone materials were rather low and possibly did not represent any high risk for adverse effects. However, other stone materials like quartzite, anorthosite and hornfels induced stronger pro-inflammatory responses than rhomb porphyry and quartz diorite in the lung cell cultures and may represent a higher hazard. Overall, this study showed that feldspar content is not a major determinant for the stone particle-induced pro-inflammatory responses (15).

The concentrations used in the exposure chamber reflect the upper OEL for total dust and respirable dust and seem relevant for exposure occurring in occupational settings. Also, in environmental settings, the concentrations of mineral PMs may be high due to abrasion of road pavement or PM pollution episodes (12, 37), although usually at significantly lower concentrations than in workplaces. In addition to the short exposure time (4 hours) used in this clinical study, it should be emphasized that adverse effects may occur at much lower concentrations in environmental settings as also more vulnerable groups are exposed.

In conclusion, the present study showed that exposure to quartz diorite significantly affects FeNO and lung function parameters with a bit delayed effect. However, quartz diorite only induced non-significant reductions in FEV_1_, FVC, and FEF25% after short-term exposure. In contrast, short-term exposure to rhomb porphyry did not significantly increase FeNO levels but was associated with significant, modest reductions in FEV_1_ and FVC. Furthermore, no significant drop in FEV_1_ was observed for the doses of methacholine administered. Which minerals and/or associated metals are involved in the observed effects remain unclear. Still, our results emphasize that the stone material should be considered when selecting asphalt materials for road construction purposes, as we cannot exclude an effect of stone materials on lung inflammation and lung function especially in vulnerable individuals.
